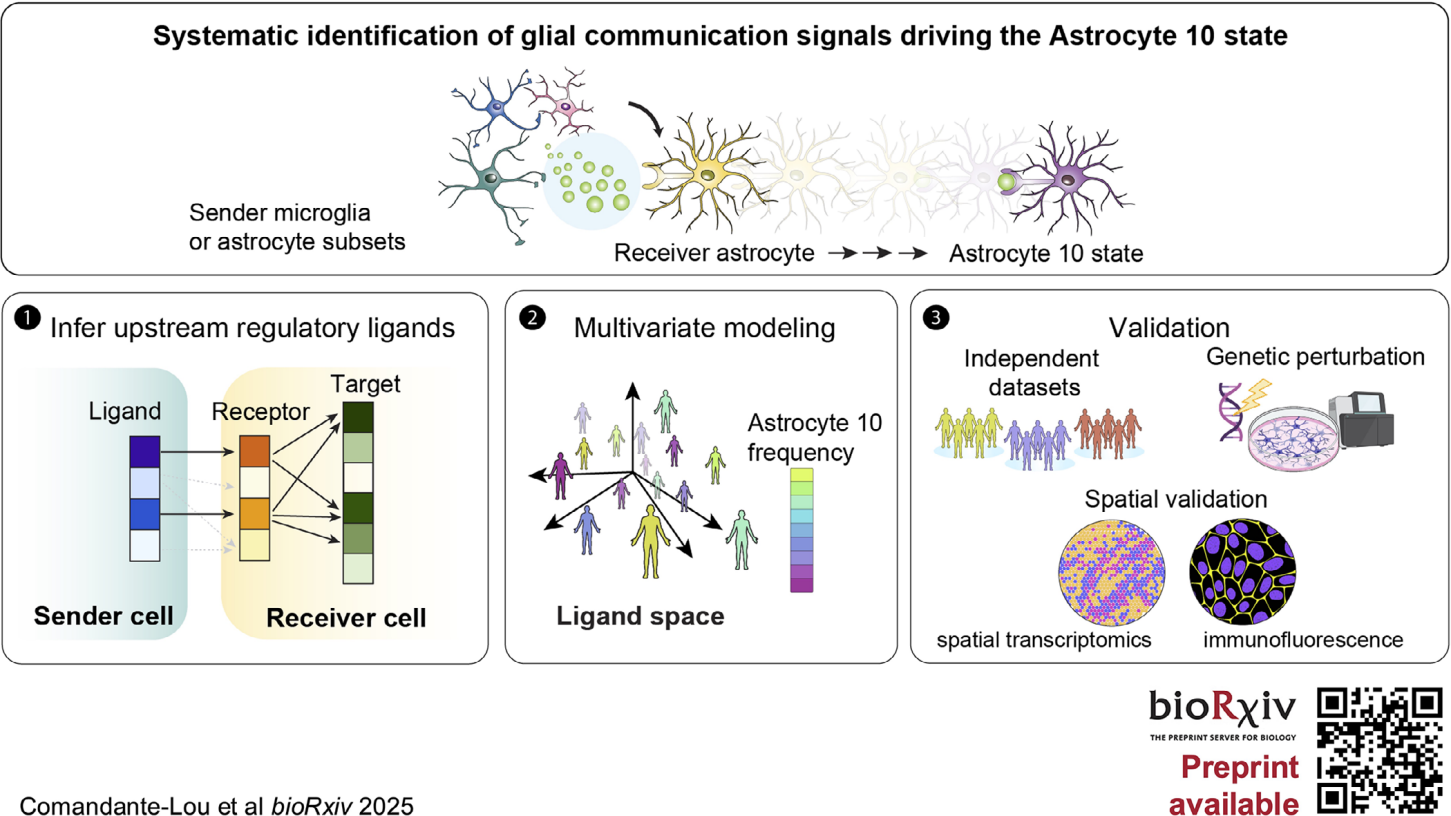# Cell‐cell signaling drives a pathologic astrocyte state contributing to cognitive decline in Alzheimer's Disease

**DOI:** 10.1002/alz70861_108184

**Published:** 2025-12-23

**Authors:** Natacha Comandante‐Lou, Tsering D Lama, Kevin W Chen, Jinglong Zhang, Bin Hu, Shuhui Liu, Sarah E Heuer, Kristen Brennand, Julie A Schneider, Lisa L. Barnes, Bin Zhang, Minghui Wang, Hongyan Zou, Roland H Friedel, Yiyi Ma, Tracy L Young‐Pearse, Aiqun Li, Masashi Fujita, David A. A. Bennett, Ya Zhang, Vilas Menon, Hans‐Ulrich Klein, Mariko Taga, Philip L. De Jager

**Affiliations:** ^1^ Center for Translational & Computational Neuroimmunology, Columbia University Irving Medical Center, New York, NY USA; ^2^ Icahn School of Medicine at Mount Sinai, New York, NY USA; ^3^ Ann Romney Center for Neurologic Diseases, Department of Neurology, Brigham and Women’s Hospital and Harvard Medical School, Boston, MA USA; ^4^ Yale School of Medicine, New Haven, CT USA; ^5^ Rush Alzheimer's Disease Center, Rush University Medical Center, Chicago, IL USA; ^6^ Harvard Medical School, Boston, MA USA

## Abstract

**Background:**

Alzheimer’s disease (AD) is marked by the coordinated emergence of disease‐associated cell states across multiple brain cell types. Among these, an astrocyte subset—Astrocyte 10 (Ast10), defined by high expression of *SLC38A2*, *SMTN*, and *CACNA1D*—has been linked to cognitive decline in AD. However, the signaling mechanisms driving Ast10’s emergence remain unclear. Here, we aim to identify the intercellular signals that promote the differentiation of astrocytes into the Ast10 state. Targeting this cell‐cell crosstalk may offer a strategy to prevent or delay AD‐associated cognitive impairment.

**Methods:**

We used NicheNet to infer ligands that regulate the Ast.10 signature based on prior knowledge of the signaling pathways. We trained a partial least squares regression model using the prioritized ligand expression to predict Ast.10 frequency across donors. To validate these ligands, we performed spatial neighborhood analysis on Visium spatial transcriptomic profiles of human cortical tissues as well as an analysis of mouse and iPSC‐derived astrocytes data.

**Results:**

Our meta‐analysis of 869 brains confirmed the strong association of Ast10 with AD and aging‐related cognitive decline. Computational modeling identified ligand‐receptor pairs predictive of Ast10 abundance across individuals. Spatial transcriptomics revealed selective colocalization of these ligands with the Ast10 signature in AD brain tissue. Genetic ablation of the receptor *PLXNB1*, a top‐ranked candidate, reduced the Ast10 transcriptional signature in astrocytes in *in vivo* and *in vitro* model systems, supporting its functional role in driving this state. Moreover, Ast10 was associated with synaptic loss and cognitive decline independently of AD pathology.

**Conclusion:**

Ast10 represents a transcriptionally distinct astrocyte state linked to cognitive decline in aging and AD. Its emergence appears to be driven by cell‐cell interactions involving *PLXNB1*. These findings suggest that Ast10 and its upstream regulators are potential therapeutic targets to mitigate cognitive decline across neurodegenerative contexts.